# The Early Recognition of Cancer of the Breast

**Published:** 1910-01-22

**Authors:** T. Crisp English

**Affiliations:** Senior Assistant Surgeon to St. George's Hospital


### Hospital Clinics.

### THE EARLY RECOGNITION OF CANCER OF THE BREAST.

By T. CRISP ENGLISH, F.R.C.S., Senior Assistant Surgeon to St. George's Hospital.

(Abstract of a Lecture delivered at St. George's Hospital.)

The increasing success which follows the surgical treatment of cancer is due to two factors—earlier operations and more thorough operations. The earlier recognition of cancer is a subject in which there is still room for considerable progress, for, although it is universally agreed that early diagnosis is essential, there is still a tendency to neglect the actual teaching and the study of the very first signs of the disease.

The student, from the beginning of his surgical career, is apt to get a wrong impression of the clinical features of cancer of the breast; too much prominence is given to the signs of the fully established disease, to the so-called "classical signs," and the illustrations in the text-books mainly concern large tumours, fungating growths, and widely spread disease of the thoracic wall. These, of course, give an excellent idea of the advanced disease, but they overshadow the early stage in which recognition is all important. "Classical signs" represent fully established disease; the outlook for patients who present these signs is bad, and operative results in these cases can only be fair. Every description of cancer of the breast should contain a special paragraph devoted to the early signs.

There is no doubt that cases now come to the surgeon at a much earlier date than formerly, but there will always, of course, be cases which are not discovered until the disease is advanced, cases in which the disease is concealed by the patient, cases in which it is watched. Concealment is largely due to a fear of operation, and to a belief that operation will be ineffectual. It is therefore of the highest importance that the public should know of the successes which follow early operations. It has been suggested that they should be taught the early signs of cancer, so that they may consult a medical man at the earliest moment; but the literal application of this suggestion would undoubtedly lead to a large number of people spending their days in fear and trembling; it would lead to introspection and to the imagination of all kinds of evil symptoms. Our obvious duty is to teach the public the importance of at once consulting a medical man even for ailments which appear trivial, and to impress upon them the success which follows early treatment.

In this lecture I shall consider the symptoms and signs which are presented in the early stages of mammary cancer. Two subjective points are noticeable; one is the absence of pain and the other the presence of a tumour.

### Early Signs and Symptoms.

*Absence of pain* is a striking feature in the great majority of cases; pain cannot be regarded as an early symptom, and so in most cases the tumour is discovered quite accidentally. This absence of pain is responsible for the overlooking of many growths in their early stages. In the few cases in which pain is complained of it is probably due to an associated mastitis, and is then of a stabbing neuralgic character, radiating from the breast to the inner side of the arm and to the scapular region. Patients who consult us on account of pain in the breast are most often not the subjects of cancer.

*The presence of a tumour*, as I have said, usually first calls the patient's attention to the existence of the disease; patients often say that they discovered the tumour when dressing or bathing. In those who are thin the tumour is usually detected early; but in stout subjects with voluminous breasts, the tumour may reach a large size before it is noticed. Superficial tumours are soon felt; those placed deeply in the breast may not be palpable by the patient even when large, and then puckering of the skin, retraction of the nipple, or pain may be the first sign to lead the patient to seek advice.

The following are the physical signs which we should look for in cases in which we suspect early carcinoma. Impaired mobility of the skin is placed first, as the clinical diagnosis depends most often upon this sign; the results of palpation are of far less value, and so are referred to later.

*Impaired mobility of the skin* overlying the tumour is by far the most important of all the physical signs in early cancer. If looked for carefully, it is found very early in cancer of the breast; it is very constant, and is of the greatest diagnostic value. The breast as a whole should be made to move over as wide an area of the chest wall as possible, and the effect should be compared with that of a similar movement on the opposite side; the slightest pulling on the skin points almost certainly to malignant disease. This pull will obviously be very slight when the growth is still small, and practice is essential for its ready detection. Halsted refers to the delicacy of the test in descriptive language: "Sometimes only the almost imperceptible suggestion of a pull, which when the very faintest possible is elicited by dislocation in one direction only; this sign, however slight, is all that is needed for diagnosis." He adds that comparison with the other side is essential, for any breast, if displaced far enough, will tug, in a way, upon the skin. This impaired mobility of the skin is due to the early involvement of the fibrous bands, the suspensory ligaments described by Sir Astley Cooper, connecting the breast to the skin. Carcinoma very early fixes one or more of these bands, and so causes that slight pulling upon the skin when the breast is moved.

*Impaired mobility of the nipple* is demonstrable in the early stage, unless the tumour is near the periphery of the breast, and corresponds to the retracted nipple of the later stages. This impairment is due to fixation of the ducts by the growth, causing an anchoring of the nipple. The sign will be detected by a careful examination of the relation of the nipple to the tumour, and by the manipulations employed to detect impaired mobility of the skin.

*Flattening of part or the whole of a normal curve* of the breast is a sign recently emphasised by Mr. G. Lenthal Cheatle, who points out that the flattening is best seen by viewing the curve from a position on a level with it, the surgeon lowering his eyes to the level of the breast.

*Alteration of the level of the nipple* may be an early sign, especially when the growth is situated at the highest or lowest part of the gland; but too much reliance must not be placed upon this sign, for one nipple may be at a different level to the other as a congenital difference, or as the result of old inflammatory trouble. The usual effect of carcinoma upon the nipple level is to raise it, and this sign, in conjunction with those already mentioned, may be of great assistance.

*Palpation*, of course, forms an important part of the examination, but palpation can seldom give certain information in early cases unless the signs already mentioned are looked for at the same time. Careful palpation with the flat of the hand will almost invariably distinguish between chronic mastitis and a tumour; but a small cyst surrounded by mastitis may feel exactly like a carcinoma. Multiple tumours, especially if occurring in both breasts, are most probably innocent, but even then one of these tumours may be an early growth.

Breast tissue should never be picked up with the fingers, for this proceeding is misleading to the surgeon and unpleasant for the patient; normal breast tissue, if picked up, will usually give the impression of a tumour. It is also of great importance that a doubtful tumour should not be manipulated too vigorously, for much harm may be done if the tumour is cancerous. Malignant tumours, and especially early ones, are often ill-defined, and palpation may even leave one in doubt as to whether there is any tumour at all. This in itself is suggestive of carcinoma, for cysts and simple tumours are easily defined. A carcinoma deeply placed behind the nipple is especially difficult to feel: in this case the signs mentioned are far more helpful than the results of palpation.

Retraction of the nipple and puckering of the skin correspond to the impaired mobility of the nipple and skin seen in the early stage; the suspensory ligaments and the ducts which were first fixed by the growth later become shortened. These signs, therefore, usually represent a comparatively advanced stage of the disease. Retraction of the nipple is a sign which has received an exaggerated importance; the time at which this sign appears depends upon the position and the nature of the growth. When the growth is situated in the central part of the breast, retraction may appear early; but when the growth is not near the nipple, retraction may be quite late.

Enlargement of the axillary glands is a late sign, and points to considerable advance of the disease, and makes the prognosis serious. I have in a previous lecture referred to Halsted's statement, based on his unique experience of large numbers of radical operations, that when no involvement of axillary glands is evident, two out of three patients are cured by operation, but when the glands are obviously diseased, three out of four die. There could be no stronger argument than this in favour of operation at the earliest possible moment.

It is obvious that in many cases of early disease the surgeon will be in doubt even after a most careful examination. In these cases the doubt must be cleared up at once by operative examination of the swelling. There can be no possible justification for delay, or for trying the effect of local applications and drugs; those who adopt this course are undertaking a grave responsibility, for which there is no excuse. Doubtful tumours usually turn out to be malignant, and therefore must be investigated at once. It is very easy to wait and watch, and patients who know not the facts of the case are usually in agreement with such a course. On the other hand, when matters are carefully and gently explained to them, patients with mammary swellings are particularly amenable to reason. Tell them plainly that they have a swelling in the breast about the nature of which it is impossible to be certain, and that it is wisest to clear the matter up at once, whilst the trouble is early, and not run the risk of allowing a serious trouble to become more advanced. There is no need to use the word "cancer" when speaking to them, for there is no more cruel word in the medical vocabulary than this.

There are several procedures recommended in the operative diagnosis of mammary tumours. These are: (1) incision on to or into the tumour, (2) excision of the tumour and macroscopical examination of the cut surface, (3) excision of the tumour and an immediate microscopical examination, "whilst the surgeon waits," (4) excision of part or whole of the tumour, and microscopical examination of hardened sections, (5) removal of a portion of the tumour for microscopical examination under local anæsthesia a few days before the proposed operation.

In practice one finds that the choice of method must depend upon the circumstances of the case. Personally I place great reliance in the older method of a limited incision into the tumour, as being less likely to cause dissemination of cancer cells than more extensive proceedings. In 90 per cent. of the cases a positive diagnosis may be made by examination of the cut surface, and in many cases it is quite sufficient to carry the incision only as far as the edge of the tumour, and not through it. In the 10 per cent. of cases where doubt still exists, the diseased area should be excised for microscopical examination. I am not in favour of an immediate microscopical report, for in difficult cases there cannot be that certainty which is the one thing desired; experienced pathologists tell one that it is often impossible to decide between chronic mastitis and early carcinoma by examination of fresh sections only; in fact, the pathologists with whom I usually work are reluctant to make these immediate examinations. Circumstances, however, may make the use of this method advisable; for instance, cases in which patients would not consent to a second operation.

In conclusion one must point out that practice and time are essential for the early detection of mammary carcinoma. Every opportunity should be taken of carefully examining tumours of the breast, and it is especially important that time should be devoted to each examination, for hurried examinations lead to little knowledge and uncertain diagnosis.

